# Pacemaker and Atrioventricular Junction Ablation in Patients With Atrial Fibrillation—A Systematic Review of Systematic Review and Meta-Analysis

**DOI:** 10.3389/fcvm.2021.587297

**Published:** 2022-01-20

**Authors:** Chi Zhang, Xi-Ying Wang, Lian Lou, Xuan Zhang, Le-Le Chen, Yu-Xiao Chen, Jian Yang

**Affiliations:** Department of Cardiology, The First Affiliated Hospital, College of Medicine, Zhejiang University, Hangzhou, China

**Keywords:** atrial fibrillation, heart failure, biventricular pacing, atrioventricular junction ablation, rate control

## Abstract

**Background:**

Cardiac resynchronization therapy (CRT) could be considered for heart failure (HF) patients with atrial fibrillation (AF) unless a potent ventricular capture strategy is conducted. However, the benefit of a pacemaker (PM; as part of CRT) in patients with AF and whether atrioventricular junction (or nodal) ablation (AVAB) can improve the prognosis of these patients compared with those treated medically to support ventricular capture are unclear.

**Methods and Results:**

Systematic reviews and meta-analyses investigating the roles of PMs and AVAB in patients with AF were obtained in a search of the PubMed, Embase, and Medline databases and then analyzed with respect to the following outcomes: mortality, left ventricular ejection fraction, and clinical findings including the New York Heart Association class, 6-min walk distance (6MWD), quality of life as assessed in a specific questionnaire, and response to CRT. The quality of the included reviews was assessed using the Assessing the Methodological Quality of Systematic Reviews 2 tool, which includes 16 items. This study was finally based on 13 systematic reviews or meta-analyses. The results showed that patients with AF have higher all-cause mortality rates compared with patients with sinus rhythm and that AVAB can reduce all-cause mortality in patients with AF. Although the functional improvement was better in sinus rhythm than in patients with AF, in the latter, AVAB increased the 6MWD and reduced the CRT nonresponse rate in patients with AF.

**Conclusion:**

Atrial fibrillation is associated with a higher all-cause mortality rate in patients with CRT implantation. AVAB, by increasing the 6MWD and survival, can improve the prognosis of these patients.

## Introduction

Cardiac resynchronization therapy (CRT) has been recommended as the class IA indication for the management of patients with prolonged QRS duration in sinus rhythm (SR) and impaired cardiac function, characterized by reduced left ventricular ejection fraction (LVEF) or the advanced New York Heart Association (NYHA) class (1). However, the benefits of CRT in patients with atrial fibrillation (AF) and heart failure (HF) are unclear, given the insufficient biventricular capture induced by the high-ventricular rate. Therefore, rate control is an essential treatment goal in AF, as decreasing the ventricular beating rate improves the cardiac ejection function and the efficacy of the implanted CRT device (2). For patients with AF with HF, the optimal ventricular rate at rest is 70–90 beats/min, according to the latest guideline of the European Society of Cardiology (ESC) (3). A ventricular rate of <70 beats/min is associated with worse outcomes in AF patients with HF and a reduced ejection fraction. This can explain the failure of the guideline-recommended target dose of beta-blockers to improve the prognosis of HF patients with AF (4, 5).

Atrioventricular junction (or nodal) ablation (AVAB) blocks the conduction of electrical signals from the upstream sinus node and atrium, thus directly decreasing the ventricular rate and increasing the ventricular pacing ratio, which is critical for the efficacy of CRT (2). AVAB is therefore a potent therapy for patients with AF who have not responded to drug treatment. The 2016 ESC guideline suggests a cardiac pacemaker (PM) accompanied by AVAB to improve the clinical symptoms of patients with drug-refractory HF and AF (6). In the guideline by Heart Rhythm Society, American College of Cardiology, and American Heart Association, AVAB combined with a permanent PM is also proposed as a rate control strategy for patients with AF while drug management is inadequate and rhythm control strategy is not feasible (7, 8). However, CRT is not an indication for AVAB in patients with AF, except in those whose ventricular rate remains high (>110 beats/min) despite pharmacological therapy. The conflicting conclusions drawn by several systematic reviews and meta-analyses reflect differences in the included primary studies. Thus, in this study, we analyzed those systematic reviews and meta-analyses to determine whether a PM and AVAB improve the clinical symptoms, cardiac function, and the occurrence of adverse events in AF and patients with HF compared with patients with SR. We also examined whether, after CRT implantation, AVAB in patients with AF with HF improves the survival rate, cardiac function, clinical symptoms, and CRT response rate.

## Methods

### Search Strategy, Study Selection, and Quality Assessment

The PubMed, Embase, and Medline databases were searched for systematic reviews and meta-analyses of PM use and AVAB in the management of patients with AF. Studies in all languages were eligible. The MeSH terms were CRT, atrioventricular junction/nodal ablation, and PM. These terms and their variants were then combined with the item AF, and an additional search was conducted. Details of the search strategy are provided in the Supplementary Material. Related reviews and the reference lists of the included reviews were also checked manually for eligible reviews and meta-analyses. All systematic reviews or meta-analyses that investigated PM (mainly CRT) implantation with or without AVAB in patients with AF and HF were included. Several conference abstracts were excluded because information on the primary research was not reported, such that the quality of that research could not be evaluated. Two reviewers (XYW and LL) independently assessed the quality of all included systematic reviews and meta-analyses using the Assessing the Methodological Quality of Systematic Reviews 2 (AMSTAR-2) tool, which contains 16 items. A discrepancy in quality assessment was resolved by consensus.

### Data Extraction and Analysis

The outcomes of interest in our analysis included mortality, LVEF, and clinical evaluations including the NYHA class, 6-min walk distance (6MWD), quality of life (QoL) assessed using a specific questionnaire, and response to CRT. Data on these outcomes were extracted from the included reviews independently. To account for overlapping primary articles included in the reviews, rather than pooling the results of the outcomes of interest, we limited our analysis to a systematic review of the results to obtain the conclusion of each included study. If the included reviews reported pooled results, the effect sizes and heterogeneity were extracted and summarized. We also report the conclusions reached in systematic reviews without pooled results.

## Results

### Results of Literature Searching and Character of Included Reviews

**Figure 1:**
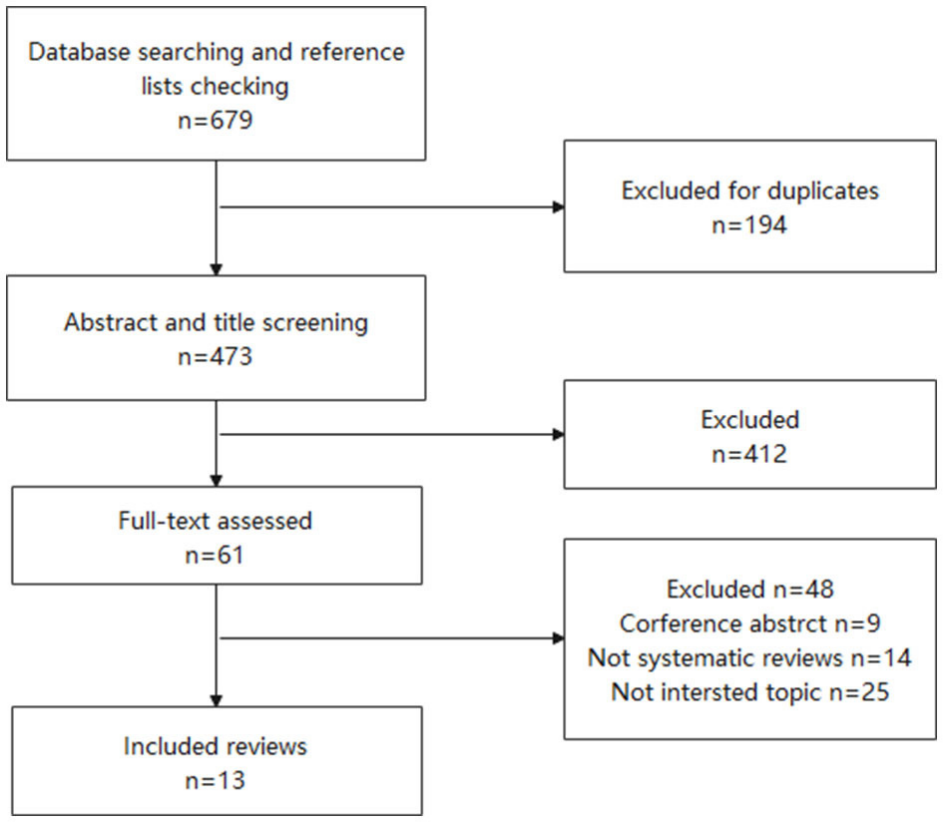
The PRISMA flowchart of the included and excluded systematic review and meta-analysis.

A search of the PubMed, Embase, and Medline databases and a manual search of the reference lists of and similar articles to the identified articles yielded 679 reports (Figure 1). After the exclusion of irrelevant reviews, 13 systematic reviews and meta-analyses (9–21) were finally included in our overview (Table 1).

**Table 1:** The main character of the included systematic reviews and meta-analyses.

**References**	**Review type**	**Design of Included studies**	**No. of patients**	**Intervention/ Comparator**	**Character of patients**	**Main conclusions**
Wood et al. (19)	Systematic Review	Unlimited types	1,181	Single-arm analysis	Patients with refractory atrial tachyarrhythmias (97% atrial fibrillation)	Exercise duration and Qol tend to be improved after ablation and pacing while the EF does not change. The mortality is also lower at 1 year.
Bradley et al. (9)	Meta-analysis	RCT	672	AF+AVAB/AF-AVAB	Patients with AF	Limited data indicate the statistical difference in the improvement in survival, cardiac function and symptoms according to the AVAB status and pacing mode.
Upadhyay et a. (16)	Meta-analysis	Prospective studies	1,164	AF/SR	Patients with SR or AF, QRS duration ≥120 ms, NYHA III/IV, LEVF ≤ 35% and treated with CRT	SR patients have greater improvement in 6WMD and MS while the AF patients have greater LVEF improvement. Mortality and NYHA improvement are similar in both groups.
Wein et al. (17)^*^	Meta-analysis	Prospective studies	1,164	AF/SR	Patients treated with CRT	SR patients have greater improvement in 6WMD and Qol while LVEF and NYHA class are similar between groups.
Wilton et al. (18)	Meta-Analysis and Systematic Review	OS	7,495	AF/SR and AF+AVAB/AF-AVAB	Patients with heart failure symptoms left ventricular (LV) ejection fraction (EF) ≤ 0.35, and QRS duration ≥120 ms, and allowed for comparisons between patients with or without AF.	AF patients have a high rate of non-response to CRT and all-cause mortality. Clinical evaluation also favors patients with SR. AVAB may improve the rate of response to CRT and all-cause mortality in AF patients.
Chatterjee et al. (10)	Meta-Analysis and Systematic Review	Unlimited types	5,632	AF+AVAB/AF-AVAB	Patients with AF treated with a pacemaker (CRT or RVP mode) with or without AVAB.	All-cause mortality, exercise duration and ejection fraction are similar between groups while subgroup analysis indicates patients with systolic dysfunction may benefit from AVAB.
Ganesan et al. (11)	Systematic Review	OS	768	AF+AVAB/AF-AVAB	Patients with QRS duration ≥120 ms and LEVF ≤ 35% treated with CRT with or without AVAB.	AVAB is related to improvement in mortality and NYHA class. And the improvement in the LVEF and 6WMD do not meet statistical significance.
Hess et al. (12)	Meta-Analysis and Systematic Review	Unlimited types	Not report	Single-arm analysis	Patients with traditional CRT indication(NYHA III/IV class, QRS duration ≥120 ms and LEVF ≤ 35%).	Limited data indicate the effect of CRT on the conversion of AF to SR.
Lopes et al. (13)	Meta-Analysis	Unlimited types	5,324	AF/SR and AF+AVAB/AF-AVAB	Patients with QRS duration ≥120 ms and LEVF ≤ 35% treated with CRT with or without AVAB.	All-cause mortality and non-response to CRT are less in the SR group, although cardiovascular death (CVD) is similar. AVAB reduce the all-cause mortality, CVD and non-response rate compared with patients without AVAB.
Yin et al. (21)	Meta-Analysis and Systematic Review	OS	1,256	AF+AVAB/AF-AVAB	Patients with HF symptoms and permanent AF, left ventricular ejection fraction (LVEF) ≤ 0.35, and QRS duration ≥120 ms	Patients with insufficient ventricular pacing (≤ 90%) tend to benefit from AVAB on the rate of mortality and non-response to CRT. Clinical evaluation and LVEF are similar in both groups.
Richard et al. (15)^#^	Systematic Review	Unlimited types	NR	AF/Non-AF	Patients with or without AF treated with CRT.	Patients treated CRT in AF rhythm have poor outcomes compared with those without AF.
Mustafa et al. (14)	Meta-Analysis and Systematic Review	OS	83,571	AF/SR and AF+AVAB/AF-AVAB	Patients with or without AF treated with CRT.	AF patients have higher all-cause mortality than SR patients. AVAB reduced the mortality in AF patients which is similar to SR patients. LVEF is similar in AF and SR groups.
Xue et al. (20)	systematic review and network meta-analysis	OS	7,896	AF+AVAB/AF-AVAB/SR	patients with left ventricular ejection fraction (LVEF) ≤ 0.35, and QRS duration ≥120 ms treat with CRT.	The mortality rate is similar in AF+AVAB and SR groups which are higher than patients in AF-AVAB group.

*NRS, nonrandomized studies; RCT, randomized controlled trials; OS, observation studies; AVAB, atrioventricular junction (or nodal) ablation; SR, sinus rhythm; RVP, right ventricular pacing; 6WMD, 6-min walk distance; MS, Minnesota score; LVEF, left ventricular ejection fraction; HF, heart failure*.

* *This meta-analysis consists of three subgroup meta-analyses according to the NYHA class, the rhythm of the heart, and QRS duration while included studies in the rhythm section are same as the meta-analysis conducted by Upadhyay et al*.

# *Subgroup analysis comparing the effect of AF rhythm and SR on CRT implantation*.

Of those 13 reviews, the systematic review conducted by Wood et al. was a single-arm analysis investigating the roles of a PM and AVAB in patients with refractory atrial tachyarrhythmias (97% AF) (19). The focus of the study of Hess et al. was the anti-arrhythmic effect of CRT in patients with new-onset or already existing AF; their study is the only review of this topic (12). Studies of patients with rapid ventricular pacing (RVP) were included in early reviews by Wood et al. and Bradley et al. (9, 19). The pacing mode in the other reviews was exclusively biventricular. In the network meta-analysis of Xue et al., the survival of patients with SR or AF treated with or without AVAB was examined (20). The systematic review by Richard et al. investigated the predictors of a response to CRT in patients in whom AF was considered a potential risk factor (15). However, most reviews do not achieve a satisfactory score in the AMSTAR-2 tool (Supplementary Table 1). Due to differences in the significance of the 16 items in the tool, we did not sum the results to achieve a total score. Although several meta-analyses indicate the quality of primary articles were evaluated, the result is not shown in the full-text or Supplementary Material on the websites of the publishers.

### All-Cause Mortality

Four reviews compared the difference in the all-cause mortality rate between the AF-PM and SR-PM groups. Three reviews (13, 14, 18) reported increased risk in the AF group, but another (16) found no statistical difference in mortality between the groups. We, therefore, reviewed the primary articles in the latter study (16) and determined that the conclusion of a similar risk was invalid after excluding a primary study (22) using a sensitivity analysis with a pooled relative risk of 1.93 (95% CI 1.12–3.30, *p* = 0.02; data not shown) and minimal heterogeneity (*I*^2^ = 20%). Six reviews and a network meta-analysis assessed the mortality of patients with AF who underwent AVAB to further control the ventricular rate. Four of the six reviews (11, 13, 14, 21) and a network meta-analysis (20) reported a lower risk, whereas the other two reviews (9, 10) found no significant difference in the mortality risk in a comparison with patients without AVAB. We examined the primary studies included in those reviews and found that the control groups of two reviews (9, 10) included those who refused PM therapy or those with paroxysmal AF. One systematic review (14) and the network meta-analysis (20) also showed a higher mortality risk in patients with AF and PM therapy without AVAB than in patients in the SR-PM group. However, the risk of all-cause mortality in AVAB-treated patients with AF was similar to that of patients with SR (Supplementary Table 2).

### Cardiovascular Mortality

Two reviews (13, 14) comparing cardiovascular mortality in AF-PM and SR-PM groups reported conflicting results, with one review (14) finding an increased risk of cardiovascular mortality in patients with AF-PM, and the other (13) showing no significant difference. In the three reviews (11, 13, 14) that assessed the effect of AVAB on cardiovascular mortality, two (11, 13) found a lower risk of cardiovascular mortality in patients with than in those without AVAB, while the third (14) found no statistical differences. None of the reviews assessed the cardiovascular mortality risk in patients with SR-PM and AF-PM according to AVAB status (Supplementary Table 3).

### Left Ventricular Ejection Fraction

Four reviews (14, 16–18) compared LVEF improvement between patients with AF and SR, with three of them (14, 17, 18) concluding that there was no significant difference. The fourth (16) found statistically greater improvement in the AF-CRT group with substantial heterogeneity reported (*I*^2^ = 97%). Four other reviews (10, 11, 14, 21) reached conflicting conclusions regarding the benefit of AVAB in improving LVEF. While three reviews (11, 14) found no significant differences in LVEF improvement, two (11, 21) reported a slight trend toward a greater improvement in the AVAB group. In the fourth review (10), different conclusions were reached after individually pooling the results of the randomized control trials (RCTs) and observational studies. However, in both cases, there was substantial heterogeneity (*I*^2^ = 97 and 78%). In addition, the subgroup analysis of patients with reduced systolic function or impaired LVEF (<45%) showed significantly greater improvement in the AVAB group (Supplementary Table 4).

### The NYHA Class and Quality of Life

Improvement in the NYHA class was assessed in three reviews (14, 16, 17). Two (14, 16) reported significantly greater improvement among patients in the SR-PM group, but the third (17) found no significant difference. A review examining the benefit of AVAB with respect to an improved NYHA class showed greater improvement among patients in the AVAB group than in the non-AVAB group. In another review, the NYHA class improvement was similar between the two groups (Supplementary Table 5).

Two reviews (16, 18) assessed the QoL, measured using the Minnesota Living with Heart Failure (MLWHF) score. Both reported a reduction in the MLWHF scores of patients with AF (mean reductions of 9.7 and 18.8 points), although the scores in those studies were slightly lower than the mean difference in the SR group. One review (10) examined the ability of AVAB to improve the MLWHF scores, without pooling the results. In the efficacy analysis based on four studies and focusing on specific symptoms, AVAB was associated with an improvement in palpitations (four studies), effort dyspnea (three studies), easy fatigue (two studies), chest discomfort (two studies), and rest dyspnea (two studies). In that same review, the 11 observational studies also indicated an improved QoL following AVAB (Supplementary Table 6).

### Six Minute-Walk Distance (6MWD)

Four reviews (14, 16–18) assessed the improvement of 6MWD in the AF-PM and SR-PM groups. Three reviews concluded that patients with SR had greater improvement in the 6MWD compared with patients with AF, although statistically significant improvement in the AF group was observed after implanting CRT. In the fourth review, characterized by substantial heterogeneity (*I*^2^ = 99%), there was no statistical difference between the AF and SR groups. Two reviews (11, 21) evaluated the effect of AVAB on 6MWD improvement, with one review (21) showing greater improvement in the AVAB group. In another review (11), improvement in the 6MWD was compared, without pooling the results; one study (23) included in the review found significant improvement in the AVAB group, whereas, in the other included study (24), there was no significant difference (Supplementary Table 7).

### Response to CRT

There were different definitions of response to CRT across the included reviews and their involved primary studies. Clinical response to CRT was defined as an increase in the 6MWD by 10%, and one class in the NYHA improvement, and survival over 6 to 12 months consistently in the three reviews (13, 18, 21) which assessed the rate of response to CRT. Also, meeting other requirements were also deemed as responders, such as survival over 6 months and a 15% reduction in the QoL scores (18, 21). The echocardiographic response was defined as a decrease in the left ventricular end-systolic volume (LVESV) by over 10% and the absolute increase in the LVEF by 5% (13). Two reviews compared the nonresponse rate in the AF and SR groups and both concluded a statistically significant higher rate of nonresponders in the patients with AF (13, 18). All of the three reviews assessed the benefit of AVAB on the response rate to CRT and two reviews (13, 18) indicated a lower rate of nonresponders in the AVAB group with statistical significance. In another review, despite no statistically significant benefit of AVAB, subgroup analysis by ventricular capture indicates patients with insufficient ventricular capture (bi-ventricular pacing (BiVP) ≤ 90%) had a lower rate of nonresponders in the AVAB group. Patients with BiVP > 90% did not show any statistical difference in the rate of the responder to CRT (Supplementary Table 8).

## Discussion

This study yielded insights into the potential role of a PM in patients with HF and the additional benefit obtained with AVAB. Despite the conflicting results of some of the examined indexes, we found that all-cause mortality is higher in patients with AF with a CRT device than in patients with SR. Although the ESC guideline (1) does not provide a recommendation regarding AVAB based on high-quality evidence, it does note that all-cause mortality is lower and the 6MWD improved in AF patients with AVAB. Therefore, while AF may be a risk factor for no response to CRT, AVAB can mitigate this adverse effect and improve the prognosis of these patients.

Patients with AF had a higher risk of all-cause mortality compared with patients with SR. A meta-analysis drew a different conclusion, but it did not hold up to a sensitivity analysis (16). The meta-analysis by Wilton et al., which included a larger number of studies, found a better survival rate in the SR group (18). The survival rate of patients with AF who underwent AVAB was similar to that of patients in the SR group, but patients without AVAB had a higher mortality rate according to two reviews, including a network meta-analysis (14, 20). However, in direct comparisons of patients with and those without AVAB, the results were conflicting. In the meta-analyses by Chatterjee et al. and Bradley et al., AVAB did not affect mortality in patients with AF (9, 25). After reviewing the articles included in the meta-analyses, we found that patients enrolled in the AVAB group included those with RVP, which may have had a detrimental effect on survival (26). Therefore, while a higher mortality rate has been reported in patients with HF with AF than in patients with SR, AVAB has a definite benefit in reducing all-cause mortality in the former. However, compared with CRT, the combined application of RVP and AVAB may result in a worse prognosis, as noted in previous studies (25, 27). Studies of cardiovascular death among patients with PM implantation and AVAB have obtained contradictory results. However, in the meta-analysis of Mustafa et al., the limited data on cardiovascular death prevented further investigation.

Most meta-analyses included in this study found similar improvements in the LVEF in patients with and those without AF. Nonetheless, the results were conflicting. Furthermore, in the only meta-analysis that favored patients with SR, the improvement compared with patients with AF was minor and there was substantial heterogeneity among the studies (MWD: 0.39 (0.22–0.55), 97%) (16). Moreover, in most of the meta-analyses, AVAB was not associated with further LVEF improvement in patients with AF. In a subgroup analysis by Chatterjee et al., a greater potential benefit of AVAB was found in patients with reduced LVEF and impaired systolic function than in those without cardiac dysfunction. A recent RCT also found a greater potential benefit of CRT and AVAB in patients with LEVF ≤ 35% (28). However, because meta-analyses of this topic are scarce, a baseline LVEF that can be used for patient selection for CRT and AVAB cannot be determined. The results of studies that evaluated cardiac function based on the NYHA class and 6MWD were also contradictory, although most indicated a trend toward a greater improvement in patients with SR. A positive effect of AVAB on the 6MWD in patients with AF was also noted. Thus, in HF patients with AF rhythm, CRT tends to provide less benefit with respect to clinical symptoms and exercise duration compared with AVAB, which can increase the duration of exercise in these patients.

The CRT response is an important prognostic index and includes both the echocardiographic and clinical responses. Three meta-analyses found that patients with AF had a lower rate of response to CRT compared with SR patients, a finding attributed to the reduced ventricular pacing ratio in patients with AF with a fast ventricular rate (29). AVAB, as a potent rate control strategy, can result in the complete ventricular capture of a PM (21). A subgroup analysis conducted by Yin et al. (21) found a greater reduction in the nonresponse rate after AVAB in patients with a ventricular pacing ratio ≤90%. Therefore, AF is a risk factor for CRT, but among patients with AF, the response rate is increased after treatment with AVAB.

In the systematic review by Hess et al. (12), CRT was associated with the conversion of persistent or permanent AF to SR, a topic not addressed in other reviews. The combined rate of rhythm conversion determined in the three included studies was 0.107 (95% CI: 0.069–0.163), with the conversions mostly occurring during the first year after implantation of the CRT device (30). Four predictors of conversion, which are related to better survival, were identified in a multivariate analysis: left ventricular diameter at end-systolic phase, left atrial diameter, QRS duration after CRT device implantation, and AVAB (30). Another study reported a lower prevalence of AF in the CRT responder group at the 6-month follow-up after device implantation than at baseline (31). This finding further supports the use of AVAB to increase both the conversion rate and CRT response rate.

In the systematic review by Mustafa et al., the outcomes of patients with CRT were not better than those with an implanted cardioverter-defibrillator, which suggests that CRT alone does not benefit patients with AF with HF. However, in most systematic reviews and meta-analyses included in this study, patients with AF treated with both the AVAB and CRT had a better prognosis than did those without AVAB. All-cause mortality was comparable between patients treated with AVAB and SR, whereas the outcomes of patients who did not undergo AVAB were poorer than those of patients with SR (14, 20). Therefore, mortality seems to be lower in patients with AF with HF treated with both the AVAB and CRT than in patients treated with CRT alone, which also indicates an important role of AVAB in the success of CRT in patients with AF.

Notably, conduction system pacing, especially His-bundle pacing (HBP), is also an important alternative pacing mode for these patients with AF who have an indication for CRT and AVAB in addition to biventricular pacing (BVP) mode. Several single-arm studies have observed the improvement of echocardiographic measurement and the NYHA class after the implantation of HBP and AVAB, while high pulmonary artery systolic pressure, high serum creatine, and low LVEF have been identified as the risk factor of poor prognosis (32, 33). But different from HBP, BVP cannot be considered as a physiological pacing mode in patients with a QRS duration <130 ms which might increase the intra- and inter-ventricular activation time in these patients (34, 35). However, HBP can still provide physiological activation sequence in these patients with narrow QRS (35) and thereby deliver more effective ventricular resynchronization (36). And sequential HBP followed by left ventricular pacing can also provide improved electrical resynchronization compared with BVP (37). HBP is also effective for patients with HF with right branch bundle block which is not an ideal indication for BVP (38). Therefore, HBP might be more preferred pacing mode for patients with AF with a lower requirement for the QRS duration (39). However, these results are drawn from the observational study which needs to be further confirmed in randomized trials. Several problems might also restrict the implementation of HBP in the current: success implantation rate, capture thresholds, sensing challenge, battery life, programming, and device algorithms (36).

Nonetheless, despite these demonstrated clinical benefits of AVAB, it cannot restore atrial systolic function, which might have a large impact on the prognosis of patients with CRT (40). Previous studies showed a greater effect of atrioventricular programming than inter-ventricular programming on cardiac function, which suggests a larger role of atrioventricular than inter-ventricular resynchronization (41, 42). However, because AVAB cannot restore atrial activity in patients with AF, its only benefit in inter-ventricular resynchronization is ensuring biventricular capture (40). Accordingly, for AF patients with HF, SR conversion by catheter ablation might be a better strategy than AVAB, as it allows atrioventricular resynchronization. This was demonstrated in a small RCT that directly compared the effect of pulmonary vein isolation with that of AVAB accompanied by CRT (43). However, patients in the trial had a narrow QRS duration and thus did not meet the indications for CRT of patients with AF according to the ESC guideline (43). In AF patients with a wide QRS duration, whether AF conversion with AVAB alone is superior to the AVAB combined with CRT requires further investigation.

In addition to the poor outcome of patients with persistent AF, a topic widely addressed in previous studies, both the intermittent and developed AF or atrial flutter are predictors of poor outcomes in patients who received CRT. While this was the conclusion reached in a subgroup analysis of the COMPANION trial (44), these patients have not been well-studied. However, in two other subgroup analyses, a history of intermittent or developed AF after CRT implantation was not associated with a poor response to CRT (45, 46). A potential explanation of these differences is the rate of BiVP, which is significantly influenced by AVAB (44). Therefore, as a history of intermittent or developed AF may have a negative effect on the success of CRT, AVAB should be strongly considered in these patients (44, 47).

## Limitations of this Study

Despite our comprehensive search of relevant systematic reviews and meta-analyses and discussion of their results, this study had several limitations. First, because we summarized the results of the included reviews, there may have been errors that resulted in bias. Second, the comparison between the patients in AF or SR rhythm restricts the implementation of RCT for reasons of different populations and the included primary articles on this are mostly observational trials, which leads to a relatively lower quality of evidence. Third, the same articles may have been included in successive meta-analyses, such that the conclusions, whether positive or negative, may have been overemphasized. Lastly, only a few meta-analyses included subgroup analyses of an effect compared with baseline, as was done by Chatterjee et al. in their analysis of LVEF and systolic function. However, this information is important for clinical decision-making and patient selection.

## Conclusion

In conclusion, AF is associated with higher all-cause mortality in patients with CRT implantation while the AVAB is noted to improve the prognosis of these patients with AF by increasing the survival rate and 6WMD.

## Data Availability Statement

The original contributions presented in the study are included in the article/Supplementary Material, further inquiries can be directed to the corresponding author.

## Author Contributions

CZ was responsible for the writing of the major parts of this overview. X-YW and LL assessed the quality of included reviews by the AMSTAR-2 tool. XZ and Y-XC took responsibility for collecting the related reviews in the reference lists and helped to discuss the results. L-LC contributed significantly to the revision of our manuscript. All authors contributed to the article and approved the submitted version.

## Funding

This study was supported by the National Natural Science Foundation of China (Project Nos. 81701365 and 81400295), the Zhejiang Provincial Natural Science Foundation of China (Nos. Z16H020002 and LY19H020008), and the Research Fund of the Health Agency of Zhejiang Province (No. 2016KYB100).

## Conflict of Interest

The authors declare that the research was conducted in the absence of any commercial or financial relationships that could be construed as a potential conflict of interest.

## Publisher's Note

All claims expressed in this article are solely those of the authors and do not necessarily represent those of their affiliated organizations, or those of the publisher, the editors and the reviewers. Any product that may be evaluated in this article, or claim that may be made by its manufacturer, is not guaranteed or endorsed by the publisher.
