# Supplementary material for: Pacemaker and Atrioventricular Junction Ablation in Patients With Atrial Fibrillation—A Systematic Review of Systematic Review and Meta-Analysis
Source: Front Cardiovasc Med. 2022 Jan 20;8:587297. doi: 10.3389/fcvm.2021.587297 (PMC8810654; doi:10.3389/fcvm.2021.587297)
Supplement: Supplementary file 1 [file Table_1.DOCX]

Searching strategy

Pubmed

(((((((((((((((((((((((((((Atrial Fibrillation[Title/Abstract]) OR (Atrial Fibrillations[Title/Abstract])) OR (Fibrillation, Atrial[Title/Abstract])) OR (Fibrillations, Atrial[Title/Abstract])) OR (Auricular Fibrillation[Title/Abstract])) OR (Auricular Fibrillations[Title/Abstract])) OR (Fibrillation, Auricular[Title/Abstract])) OR (Fibrillations, Auricular[Title/Abstract])) OR (Persistent Atrial Fibrillation[Title/Abstract])) OR (Atrial Fibrillation, Persistent[Title/Abstract])) OR (Atrial Fibrillations, Persistent[Title/Abstract])) OR (Fibrillation, Persistent Atrial[Title/Abstract])) OR (Fibrillations, Persistent Atrial[Title/Abstract])) OR (Persistent Atrial Fibrillations[Title/Abstract])) OR (Familial Atrial Fibrillation[Title/Abstract])) OR (Atrial Fibrillation, Familial[Title/Abstract])) OR (Atrial Fibrillations, Familial[Title/Abstract])) OR (Familial Atrial Fibrillations[Title/Abstract])) OR (Fibrillation, Familial Atrial[Title/Abstract])) OR (Fibrillations, Familial Atrial[Title/Abstract])) OR (Paroxysmal Atrial Fibrillation[Title/Abstract])) OR (Atrial Fibrillation, Paroxysmal[Title/Abstract])) OR (Atrial Fibrillations, Paroxysmal[Title/Abstract])) OR (Fibrillation, Paroxysmal Atrial[Title/Abstract])) OR (Fibrillations, Paroxysmal Atrial[Title/Abstract])) OR (Paroxysmal Atrial Fibrillations[Title/Abstract])) AND (((((((((((((((Cardiac Resynchronization Therapy[Title/Abstract])) OR (Resynchronization Therapy, Cardiac[Title/Abstract])) OR (Therapy, Cardiac Resynchronization[Title/Abstract])) OR (Cardiac Resynchronization Pacing Therapy[Title/Abstract])) OR (Resynchronization Pacing Therapy, Cardiac[Title/Abstract])) OR (Cardiac Resynchronization[Title/Abstract])) OR (Resynchronization, Cardiac[Title/Abstract])) OR (Atrio-Biventricular Pacing[Title/Abstract])) OR (Atrio Biventricular Pacing[Title/Abstract])) OR (Pacing, Atrio-Biventricular[Title/Abstract])) OR (Biventricular Pacing[Title/Abstract])) OR (Pacing, Biventricular[Title/Abstract])) OR ((((((((((Pacemaker[Title/Abstract]) OR (Artificial Pacemaker[Title/Abstract])) OR (Artificial Pacemakers[Title/Abstract])) OR (Pacemakers, Artificial[Title/Abstract])) OR (Cardiac Pacemaker, Artificial[Title/Abstract])) OR (Artificial Cardiac Pacemaker[Title/Abstract])) OR (Artificial Cardiac Pacemakers[Title/Abstract])) OR (Cardiac Pacemakers, Artificial[Title/Abstract])) OR (Pacemaker, Artificial Cardiac[Title/Abstract])) OR (Pacemakers, Artificial Cardiac[Title/Abstract]))) OR (((((((Pacing[Title/Abstract]) OR (Artificial Cardiac Pacing[Title/Abstract])) OR (Artificial Cardiac Pacings[Title/Abstract])) OR (Cardiac Pacings, Artificial[Title/Abstract])) OR (Pacing, Artificial Cardiac[Title/Abstract])) OR (Pacings, Artificial Cardiac[Title/Abstract])) OR (Pacing, Cardiac, Artificial[Title/Abstract])) OR (atrioventricular junction ablation[Title/Abstract])) OR (atrioventricular nodal ablation[Title/Abstract]))) AND (("Systematic Review" [Publication Type]) OR "Meta-Analysis" [Publication Type]))

Embase and Medline are searched in the manner similar to searching the Pubmed with individual styles.

Table 1. The score of included reviews assessed via AMSTAR-2 tool.

| ***Author,***  ***year*** | ***Xue***  ***2019*** | ***Mustafa 2019*** | ***Yin 2014*** | ***Lopes 2014*** | ***Ganesan 2012*** | ***Chatterjee 2012*** | ***Wilton 2011*** | ***Wein 2010*** | ***Upadhyay 2008*** | ***Bradley 2007*** | ***Rickard 2016*** | ***Hess 2013*** | ***Wood 2000*** |
| --- | --- | --- | --- | --- | --- | --- | --- | --- | --- | --- | --- | --- | --- |
| ***PICO*** | *Yes* | *Yes* | *Yes* | *Yes* | *Yes* | *Yes* | *Yes* | *Yes* | *Yes* | *Yes* | *Yes* | *Yes* | *Yes* |
| ***Prior***  ***Protocol*** | *No* | *p-yes* | *No* | *No* | *No* | *No* | *No* | *No* | *No* | *No* | *No* | *No* | *No* |
| ***Inclusion reasons for RCTs or NRSI*** | *Yes* | *Yes* | *Yes* | *No* | *Yes* | *Yes* | *Yes* | *No* | *Yes* | *No* | *No* | *No* | *Yes* |
| ***Search strategy*** | *p-yes* | *p-yes* | *p-yes* | *p-yes* | *p-yes* | *p-yes* | *p-yes* | *p-yes* | *p-yes* | *No* | *p-yes* | *No* | *p-yes* |
| ***Duplicate***  ***selection*** | *Yes* | *Yes* | *No* | *Yes* | *Yes* | *No* | *Yes* | *Yes* | *No* | *No* | *No* | *No* | *No* |
| ***Duplicate***  ***extraction*** | *Yes* | *Yes* | *Yes* | *No* | *No* | *Yes* | *No* | *Yes* | *Yes* | *No* | *No* | *No* | *No* |
| ***List of excluded studies*** | *No* | *No* | *No* | *No* | *No* | *No* | *No* | *No* | *No* | *No* | *No* | *No* | *No* |
| ***Detail of included studies*** | *p-yes* | *Yes* | *p-yes* | *No* | *p-yes* | *No* | *No* | *p-yes* | *p-yes* | *Yes* | *p-yes* | *p-yes* | *p-yes* |
| ***Assessing the RoB*** | *p-yes* | *Yes* | *Yes* | *NR** | *Yes* | *NR** | *Yes* | *NR** | *NR** | *NR** | *NR** | *No* | *No* |
| ***Sources of funding*** | *No* | *No* | *No* | *No* | *No* | *No* | *No* | *No* | *No* | *No* | *No* | *No* | *No* |
| ***Appropriate***  ***combination*** | *Yes* | *Yes* | *Yes* | *No* | *Yes* | *Yes* | *Yes* | *No* | *No* | *Yes* | *No^##^* | *No* | *No* |
| ***Assess the potential impact of RoB*** | *No* | *Yes* | *No* | *No* | *No* | *---^#^* | *Yes* | *---^#^* | *---^#^* | *---^#^* | *No^##^* | *No* | *No* |
| ***Account for RoB*** | *No* | *No* | *No* | *No* | *Yes* | *---^#^* | *Yes* | *---^#^* | *---^#^* | *---^#^* | *---^#^* | *No* | *No* |
| ***Explanation for heterogeneity*** | *No* | *Yes* | *Yes* | *NR** | *Yes* | *No* | *Yes* | *No* | *Yes* | *Yes* | *No^##^* | *No* | *No* |
| ***Publication bias*** | *No* | *Yes* | *Yes* | *Yes** | *No* | *No* | *No* | *No* | *No* | *Yes* | *No^##^* | *No* | *No* |
| ***Conflict of interest*** | *Yes* | *Yes* | *No* | *Yes* | *No* | *Yes* | *No* | *No* | *No* | *No* | *Yes* | *No* | *No* |

*Claimed to be done with no details reported in the reviews.

**Another meta-analysis comparing the biventricular vs. right ventricular pacing mode

^#^For reasons of lack of details of RoB.

*^##^*No meta-analysis conducted

Table 2. Summary of the compare of all-cause mortality for pacing therapy and atrioventricular junction (or nodal) ablation in AF and SR patients in included reviews.

| *Intervention vs control* | *Review* | *Reported Effect estimate（95% CI）, I^2^* | *interpretation* |
| --- | --- | --- | --- |
|  |  |  |  |
| *AF+pacing vs SR +pacing* | ***Upadhyay 2008*** | RR:1.57(0.87,2.81),47.5% | No statistical difference |
|  | ***Wilton 2011*** | RR:1.50(1.08,2.09),61.5% | *Increased risk in AF group* |
|  | ***Lopes 2014*** | OR:1.69(1.20,2.37),54% | *Increased risk in AF group* |
|  | ***Mustafa 2019*** | OR:1.472(1.301,1.664),29.9% | *Increased risk in AF group* |
| AF+pacing+A-VAB vs AF+pacing-AVAB | ***Bradley 2007*** | RR:1.18(0.26,5.22),NR | No statistical difference |
|  | ***Chatterjee 2012*** | RR:1.05(0.29,3.85),NR | No statistical difference |
|  | ***Ganesan 2012*** | RR:0.42(0.26,0.68),0% | *Lower risk in AVAB group* |
|  | ***Lopes 2014*** | OR:0.42(0.22,0.80), 0% | *Lower risk in AVAB group* |
|  | ***Yin 2014*** | RR:0.63(0.42,0.96),48% | *Lower risk in AVAB group* |
|  | ***Mustafa 2019*** | OR:0.485(0.247,0.952),54% | *Lower risk in AVAB group* |
|  | ***Xue 2019*** | HR:0.50(0.23,1.10),60.4%* | No statistical difference |
|  |  | HR:0.64(0.46,0.91)^#^ | *Lower risk in AVAB group* |
| *AF+pacing+A-VAB vs SR-pacing* | ***Mustafa 2019*** | OR:1.243(0.911,1.696),NR | No statistical difference |
|  | ***Xue 2019*** | HR:1.00(0.73,1.40) ^#^ | No statistical difference |
| *AF+pacing-A-VAB vs SR-pacing* | ***Mustafa 2019*** | OR:1.492(1.250,1.654),P=0.000 | *Lower risk in SR group* |
|  | ***Xue 2019*** | HR:0.63(0.53,0.75) ^#^ | *Lower risk in SR group* |

AF: atrial fibrillation, SR: sinus rhythm, AVAB: atriovetricular junction (or nodal) ablation

*calculated by direct meta-analysis, ^#^ calculated by network meta-analysis.

Table 3. Summary of the compare of cardiovascular mortality for pacing therapy and atrioventricular junction (or nodal) ablation in AF and SR patients in included reviews.

| *Intervention vs control* | *Review* | *Reported Effect estimate（95% CI）, I^2^* | *interpretation* |
| --- | --- | --- | --- |
| *AF +AVAB vs AF-AVAB* | ***Lopes 2014*** | OR:0.39(0.20,0.75),0% | *Lower risk in AVAB group* |
|  | ***Mustafa 2019*** | OR: 0.764(0.580,1.005),NR | No statistical difference |
|  | ***Ganesan 2012*** | RR:0.44(0.24,0.81),0% | *Lower risk in AVAB group* |
| *AF+pacing vs SR +pacing* | ***Lopes 2014*** | OR:1.36(0.92,2.01),33% | No statistical difference |
|  | ***Mustafa 2019*** | OR: 1.857(1.390,2.554),62% | *Increased risk in AF group* |

Table 4. Summary of the compare of the change in left ventricular ejection fraction for pacing therapy and atrioventricular junction (or nodal) ablation in AF and SR patients in included reviews.

| *Intervention vs control* | *Review* | *Reported Effect estimate%（95% CI）, I^2^* | *Interpretation* |
| --- | --- | --- | --- |
| *AF+pacing vs SR +pacing* | ***Upadhyay 2008*** | WMD:0.39(0.22,0.55),97% | *Statistically greater change in AF group* |
|  | ***Wein 2010*** | SMD:0.38(-0.53,1.28),NR | No statistical difference |
|  | ***Wilton 2011*** | WMD:0.00(-0.02,0.02),NR | No statistical difference |
|  | ***Mustafa 2019*** | SMD:0.256(-1.072,1.585),99% | No statistical difference |
| *AF +AVAB vs AF-AVAB* | ***Ganesan 2012*** | MD:6.1(-3.5,15.8),94% | No statistical difference |
|  | ***Yin 2014*** | MD:1.39(-2.26,5.04),81% | No statistical difference |
|  | ***Chatterjee 2012*** | MD:1.0(-3.7,5.7),97%* | No statistical difference**** |
|  |  | MD:4.8(2.01,7.58),78%^#^ | *Statistically greater change in AVAB group^##^* |
|  | ***Mustafa 2019*** | SMD:-1.148(-5.993,3.696),NR | No statistical difference |

*calculated in randomized, controlled trials or prospective cohort studies (efficacy analysis). **When stratified by systolic function, patients with reduced systolic function have a statistically significant improvement in EF (4%; 95% CI, 3.1– 4.9) with minimal heterogeneity (I^2^=0%). In contrast, patients with normal systolic function have no significant change (2.07%; 95% CI, 8.0 to 3.8) with substantial heterogeneity (I^2^=95%).

^#^calculated in obeservational prospective and retrospective cohort studies (effective analysis).

^##^ When stratified by EF, patients with EF<45% have significant improvement in EF (7.44%; 95% CI, 5.4 –9.5) with minimal heterogeneity (I^2^=0%). Patients with EF>45% show no significant improvement (1.94%; 95% CI, 2.9% to 6.8%) with substantial heterogeneity (I^2^=88%).

Table 5. Summary of the compare of the change in NYHA class for pacing therapy and atrioventricular junction (or nodal) ablation in AF and SR patients in included reviews.

| *Intervention vs control* | *Review* | *Reported Effect estimate（95% CI）, I^2^* | *Interpretation* |
| --- | --- | --- | --- |
| *AF+pacing vs SR +pacing* | ***Upadhyay 2008*** | WMD:-0.06(-0.08,-0.04),NR | *Statistically greater change in SR group* |
|  | ***Wein 2010*** | SMD:-0.32(-1.40,0.77),NR | No statistical difference |
|  | ***Mustafa 2019*** | SMD:-0.604(-1.012,-0.195),94%* | *Statistically lower class in SR group* |
| *AF+pacing+A-VAB vs AF+pacing-AVAB* | ***Ganesan 2012*** | MD:0.34(0.13, 0.56),59% | *Statistically greater change in AVAB group* |
|  | ***Yin 2014*** | MD:0.1(-0.1,0.3),NR | No statistical difference |

*represent the difference of NYHA class at the end of follow-up rather than the difference of change in the NYHA class during follow-up.

Table 6. Summary of the compare of the change in Minnesota Living with Heart Failure score for pacing therapy and atrioventricular junction (or nodal) ablation in AF and SR patients in included reviews.

| *Intervention vs control* | *Review* | *Reported Effect estimate（95% CI）, I^2^* | *Interpretation* |
| --- | --- | --- | --- |
| *AF+pacing vs SR +pacing* | ***Upadhyay 2008*** | WMD:-3.94(-4.54,-3.35),NR | *Statistically greater change in SR group* |
|  | ***Wilton 2011*** | MD:-4.1(-6.6,-1.7),NR | *Statistically greater change in SR group* |

Table 7. Summary of the compare of the change in six-minutes walk distance (6MWD) for pacing therapy and atrioventricular junction (or nodal) ablation in AF and SR patients in included reviews.

| *Intervention vs control* | *Review* | *Reported Effect estimate（95% CI）, I^2^* | *Interpretation* |
| --- | --- | --- | --- |
| *AF+pacing vs SR +pacing* | ***Upadhyay 2008*** | WMD:-38.01(-34.06,-41.95),NR | *Statistically greater change in SR group* |
|  | ***Wein 2010*** | SMD:-1.67(-0.55,-2.79),NR | *Statistically greater change in SR group* |
|  | ***Wilton 2011*** | MD:-14.1(0.0,-28.2),NR | *Statistically greater change in SR group* |
|  | ***Mustafa 2019*** | SMD: 0.699(-0.430,1.828),99% | No statistical difference |
| *AF+AVAB vs AF-AVAB* | ***Yin 2014*** | MD:16.72(13.42,46.85),NR | *Statistically greater change in AVAB group* |

Table 8. Summary of the compare of the rate of non-response to CRT for CRT therapy and atrioventricular junction (or nodal) ablation in AF and SR patients in included reviews.

| *Intervention vs control* | *Review* | *Reported Effect estimate（95% CI）, I^2^* | *Interpretation* |
| --- | --- | --- | --- |
| *AF+pacing vs SR +pacing* | ***Wilton 2011*** | RR:1.32(1.12,1.55),0% | *Statistically higher rate in AF group* |
|  | ***Lopes 2014*** | OR:1.41(1.15,1.73),0% | *Statistically higher rate in AF group* |
| *AF+AVAB vs AF-AVAB* | ***Wilton 2011*** | RR: 0.40 (0.28, 0.58),NR | *Statistically lower rate in AVAB group* |
|  | ***Lopes 2014*** | OR: 0.31(0.10---0.97), 72% | *Statistically lower rate in AVAB group* |
|  | ***Yin 2014*** | RR:0.67(0.43,1.04),73%* | No statistical difference |

*Subgroup analysis indicates that patients with insufficient BiVP (<90%) have lower rate of non-response to CRT (RR: 0.41, 95% CI:0.31-0.54, p<0.00001, I^2^=0,), while patients with sufficient BiVP (>90%) have similar rate of non-response(RR:0.97 95% CI:0.72-1.32, p=0.87).
